# Neuroendocrine Determinants of Polycystic Ovary Syndrome

**DOI:** 10.3390/ijerph19053089

**Published:** 2022-03-06

**Authors:** Anna Szeliga, Ewa Rudnicka, Marzena Maciejewska-Jeske, Marek Kucharski, Anna Kostrzak, Marta Hajbos, Olga Niwczyk, Roman Smolarczyk, Blazej Meczekalski

**Affiliations:** 1 Department of Gynecological Endocrinology, Poznan University of Medical Sciences, 60-535 Poznan, Poland; annamaria.szeliga@gmail.com (A.S.); marzena@jeske.pl (M.M.-J.); ankos30@op.pl (A.K.); olga.niwczyk@gmail.com (O.N.); 2 Department of Gynecological Endocrinology, Medical University of Warsaw, 00-315 Warsaw, Poland; ewa.rudnicka@poczta.onet.pl (E.R.); marekkucharski@outlook.com (M.K.); martahajbos@gmail.com (M.H.); rsmolarczyk@poczta.onet.pl (R.S.)

**Keywords:** PCOS, KNDy, phoenixin, kisspeptin, neurokinin b

## Abstract

Polycystic ovary syndrome (PCOS) is the most common endocrine disorder in women and a major cause of anovulatory infertility. A diagnosis of PCOS is established based the presence of two out of three clinical symptoms, which are criteria accepted by the ESHRE/ASRM (European Society of Human Reproduction and Embryology/American Society for Reproductive Medicine). Gonadotropin-releasing hormone (GnRH) is responsible for the release of luteinizing hormone, and follicle stimulating hormone from the pituitary and contributes a leading role in controlling reproductive function in humans. The goal of this review is to present the current knowledge on neuroendocrine determinations of PCOS. The role of such neurohormones as GnRH, and neuropeptides kisspeptin, neurokinin B, phoenixin-14, and galanin is discussed in this aspect. Additionally, different neurotransmitters (gamma-aminobutyric acid (GABA), glutamate, serotonin, dopamine, and acetylcholine) can also be involved in neuroendocrine etiopathogenesis of PCOS. Studies have shown a persistent rapid GnRH pulse frequency in women with PCOS present during the whole ovulatory cycle. Other studies have proved that patients with PCOS are characterized by higher serum kisspeptin levels. The observations of elevated serum kisspeptin levels in PCOS correspond with the hypothesis that overactivity in the kisspeptin system is responsible for hypothalamic-pituitary-gonadal axis overactivity. In turn, this causes menstrual disorders, hyperandrogenemia and hyperandrogenism. Moreover, abnormal regulation of Neurokinin B (NKB) is also suspected of contributing to PCOS development, while NKB antagonists are used in the treatment of PCOS leading to reduction in Luteinizing hormone (LH) concentration and total testosterone concentration. GnRH secretion is regulated not only by kisspeptin and neurokinin B, but also by other neurohormones, such as phoenixin-14, galanin, and Glucagon-like peptide-1 (GLP-1), that have favorable effects in counteracting the progress of PCOS. A similar process is associated with the neurotransmitters such as GABA, glutamate, serotonin, dopamine, and acetylcholine, as well as the opioid system, which may interfere with secretion of GnRH, and therefore, influence the development and severity of symptoms in PCOS patients. Additional studies are required to explain entire, real mechanisms responsible for PCOS neuroendocrine background.

## 1. Introduction

Polycystic ovary syndrome (PCOS) is the most common endocrine disorder in women and a major cause of anovulatory infertility [1].

A diagnosis of PCOS is established based on criteria accepted by the ESHRE/ASRM in Rotterdam in 2003. To establish a diagnosis of PCOS, the presence of two out of three clinical symptoms is required: oligo- or anovulation, biochemical or clinical hyperandrogenism (hirsutism, acne, or androgenetic alopecia), and characteristics of a polycystic ovarian morphology (PCOM) seen on ultrasound examination of the ovaries (an ovary with at least 20 follicles and having a diameter of 2 to 9 mm or a volume of at least 10 cm^3^) [2].

PCOS is not a stand-alone endocrine disease, but a complex of symptoms [3] resulting from many genetic and environmental factors including intrauterine development disturbances, androgen excess exposure in utero, low birth weight, early pubarche, increased risk of obesity, metabolic syndrome, and type 2 diabetes [4]. Exposure to advanced glycation end-products (AGEs), often found in thermally processed foods, as well as diets high in protein and low in carbohydrates, stimulate insulin resistance. Exposure to advanced AGEs, which are contained in thermally processed foods and diets high in protein and low in carbohydrates, stimulates insulin resistance [5,6]. Increased levels of circulating insulin play a crucial role in the development of PCOS [7]. Excess insulin, acting synergistically with LH, stimulates ovarian theca cells to increase androgen production, and this excess of androgens inhibits the production of hepatic sex hormone binding globulin. An excess of androgens in the ovaries, in turn, contributes to insulin resistance. Thus, a vicious cascade of events arises; excess insulin stimulates further androgen production in the ovaries and a reduction in the production of sex hormone binding globulin (SHBG) in the liver.

Patients with PCOS demonstrate an increased level of circulation luteinizing hormone, which stimulates androgen secretion by ovarian theca cells [8,9].

Increased GnRH leads to a higher frequency of LH pulsation, stimulation of LH-mediated androgen production, and disruption of follicle development. The resulting chronic anovulation is due to the relatively low level of Follicle-stimulating hormone (FSH) that occurs secondary to the altered GnRH release pattern. Follicles exhibit relative resistance to the follicle-stimulating hormone at the ovarian level. It is possible, however, that this is related to the higher quantities of anti-Müllerian hormone released by the larger cohort of preantral follicles.

The purpose of our research is to establish a relationship between polycystic ovarian syndrome and neuroendocrine determinants. We focus on KNDy neurons, a group which express Kisspeptin, Neurokinin B, and Dynorphin A, produced in the hypothalamus. KNDy play a key role in regulating pulsatile secretion of GnRH and as mediators of steroid hormone negative feedback.

## 2. Methods

A systematic literature search for relevant English language publications published until October 2021 was conducted in several major databases, including PubMed and ScienceDirect. Authors investigated the available data from clinical studies, review articles, and meta-analyses following Medical Subject Headings (MeSH) terms, alone or in combination: PCOS, Polycystic ovary syndrome, GnRH, Gonadotropin-releasing hormone, Phoenixin, Galanin, Kisspeptin, Neurokinin B, Dynorphin A, KNDy neurons, and neurohormones. Moreover, reference lists of included articles were manually screened to identify additional studies.

## 3. GnRH Secretion in PCOS Patients

In the female body, the gonadotropin releasing hormone (GnRH) is involved in the secretion of LH and FSH from the pituitary gland. These hormones play a significant role in the functioning of the female reproductive system. In the physiological state, GnRH is released in pulses, with the lowest frequency during the luteal phase due to feedback inhibition by progesterone [10]. Studies have shown a persistent rapid GnRH pulse frequency in women with PCOS present during the whole ovulatory cycle [11]. This imbalance is mainly the cause of decreased sensitivity in the pulse generating cycle to progesterone and estrogen’s feedback inhibition [10]. Although the pathomechanism leading to this disorder remains poorly understood, some studies links reduced sensitivity to progesterone negative feedback with elevated androgen levels, two common features observed in PCOS [12]. Beyond those already mentioned, the various neuroendocrine and hormone influences on GnRH will be discussed in this article.

Rapid GnRH pulsation frequency promotes LH secretion, which leads to elevated serum LH levels and FSH levels typically in the lower follicular range. An increased LH to FSH ratio is common seen in 55–75% of women diagnosed with PCOS [1]. This hormonal imbalance leads to anovulatory cycles caused by insufficient FSH whose role it is to recruit and stimulate growth and maturation of ovarian follicles. Insufficient FSH levels disrupt the selection of a dominant follicle, which leads to accumulation of small (2–9 mm) pre-antral follicles [13]. Furthermore, anti-Müllerian hormone (AMH), which is normally secreted by antral follicles, will be elevated in PCOS and will decrease the sensitivity of growing follicles to FSH [14]. In a physiological ovarian cycle, only one dominating follicle is sensitive to the surge in luteinizing hormone at its peak. In a group of women with PCOS associated anovulation, a premature responsiveness was shown in small antral follicles to elevated levels of LH, which resulted in those follicles achieving early terminal differentiation [15]. This nascent endpoint results in the characteristic ovarian morphology seen on ultrasonography (USG) and constitutes one of the three Rotterdam diagnostic criteria (2003) for PCOS. Polycystic ovarian morphology (PCOM) is defined as enlarged ovaries (>10 cc) with at least 20 small peripherally allocated follicles and without evidence of corpora lutea nor dominant follicle [16]. Elevated levels of LH will also stimulate androgen synthesis in ovarian theca cells leading to hyperandrogenemia, which is responsible for hirsutism, acne and alopecia-common complaints at presentation in women with PCOS [17]. Hyperandrogenemia may also trigger faster GnRH pulsation frequency.

## 4. KNDy Neurons

Pulsatile release of GnRH is essential for pubertal maturation, regulation of menstrual cycles, and fertility. The pioneer studies in the early 1970s documented the fundamental role of pulsatile GnRH secretion in controlling the function of the human reproductive axis [18,19]. Investigation into the neural mechanisms and integrated signals affecting the hypothalamic-pituitary-gonadal (HPG) axis is ongoing to this day. Despite years of study into GnRH pulse generation, its underlying pulse generation mechanism remained unclear until the beginning of the 21st century. The discovery of kisspeptin was a breakthrough in understanding the GnRH neuronal network and the key role played by KNDy neurons in regulating pulsatile secretion of GnRH as so-called “GnRH pulse generator” [20,21].

**Figure 1:**
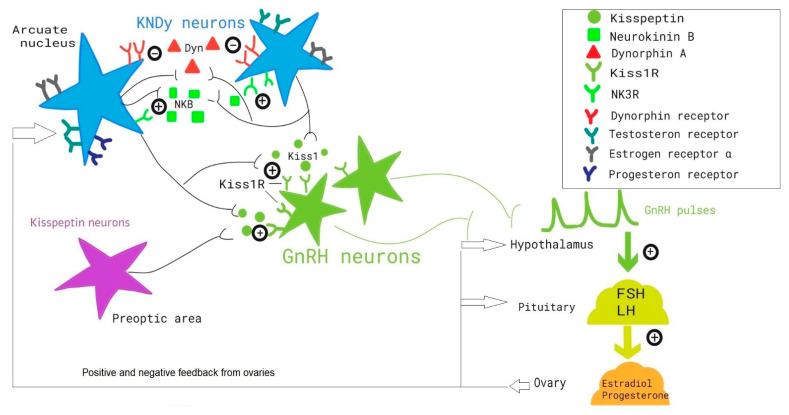
Schematic representation of the role of KNDy neurons in the regulation of the hypothalamic–pituitary–ovarians axis.

KNDy neurons are a group of neurons located in the infundibular nucleus of hypothalamus (IFN). These neurons present the ability to co-express kisspeptin, neurokinin B (NKB), and dynorphin [22,23]. The KNDy model proposes that the stimulatory role of NKB and the inhibitory role of dynorphin is crucial for the coordination of kisspeptin secretion in regulating the pulsatile release of GnRH and subsequent downstream gonadotropin release (Figure 1).

Kisspeptin acts directly on GnRH neurons through the G-protein-coupled receptors 54 (GPR54) to drive the secretion of GnRH. It is unlikely that kisspeptin itself affects KNDy neurons as no GPR54 have been found on their surface [24]. Most kisspeptin cells in the IFN co-express dynorphin and NKB which modulate kisspeptin secretion in an autocrine manner [25]. Neurokinin B belongs in the tachykinin family of peptides and interacts mainly with the neurokinin-3 receptor (NK3R). KNDy neurons express NK3R which supports the argument for autosynaptic and direct initial signaling from NKB to kisspeptin release [26]. Dynorphin acts via K opioid receptors (KOR) on both KNDy and GnRH neurons. Shortly after GnRH pulse onset, dynorphin is released in the KNDy network, in order to inhibit kisspeptin, and remains throughout the duration of the pulse. In contrast, dynorphin is released directly onto GnRH secreting neurons in order to terminate each pulse [27].

Despite the pivotal role of GnRH in the human reproductive axis, GnRH neurons themselves are not able to integrate external factors which influence their secretion. Studies suggest that KNDy neurons form signaling pathways to transduce these external signals [22].

Estradiol modulates GnRH and LH release across the menstrual cycle with both positive and negative feedback. The exact mechanism of modulation was unknown for many years as estrogen does not directly impact GnRH neurons due to their lack of estrogen receptor alpha expression [28]. Recent studies brought to light the role of kisspeptin and NKB as signal regulators. Estrogen suppresses the release of kisspeptin and NKB which results in the decrease of GnRH and LH levels. In contrast, the preovulatory LH surge in late follicular phase is triggered by positive feedback from estrogen. The exact mechanism of this feedback cycle and the precise role of KNDy neurons of the IFN and medial preoptic area KISS1 cells is still the subject of study [29]. Progesterone is the main contributing factor responsible for a reduction in LH pulse frequency in the luteal phase. The inhibitory effects of progesterone is mediated by the progesterone receptor (PR) present on KNDy neurons. There is evidence to show that negative feedback from progesterone is transmitted through dynorphin signaling in that progesterone increases dynorphin output [30]. Moreover, studies have pointed to KNDy neurons as having a key role in mediating the negative feedback of androgens on gonadotropin secretion. It has been shown that decreases in Kiss1 mRNA in the hypothalamus are liked to increases in testosterone [31].

The role of various internal and external signals (such as physical and psychological stress, nutrition, and other neurotransmitters) on the coordination on KNDy neurons still ongoing.

## 5. Kisspeptin and PCOS

KNDy neurons are found in the arcuate nucleus of the hypothalamus and express kisspeptin, neurokinin B, and dynorphin. Their localization in the hypothalamus is strategic in its proximity to the origin of GnRH secretion.

Kisspeptin is a relatively new neuropeptide and is regarded as an integral and strong positive stimulator of GnRH’s pulsatile secretion [32]. Considering the strong neuroendocrine foundation of PCOS, it is important to consider the role of kisspeptin in its pathophysiology [33].

Panidis et al. [34] were the first to compare serum kisspeptin levels in patients with PCOS and healthy controls. They found that serum kisspeptin levels negatively correlate with BMI, free androgen index, and indices of insulin resistance. Subsequent studies have confirmed the observation of elevated kisspeptin levels seen in PCOS. In a literature review by Tang et al. [35] conducted in July 2018, twelve studies were found which considered serum kisspeptin levels in PCOS. Eight of the twelve studies found significantly higher serum levels in patients with PCOS.

Significant developments have been made in recent years considering kisspeptin and its role as a potential diagnostic or prognostic marker of PCOS. Kisspeptin has largely been accepted as a diagnostic marker when needing to distinguish between functional hypothalamic amenorrhea and PCOS. In addition to Kisspeptin, 5-alpha reductase activity, leptin, insulin-like factor 3 (INSL3), and inhibin B are also markers of potential significance [36].

Liu et al. [37] have proposed a broader hypothesis in that kisspeptin can be regarded as an independent biomarker of PCOS. This proposition was made on the basis of a large meta-analysis (1282 participants: 699 patients and 583 controls) which noted that serum kisspeptin levels were higher in patients with PCOS compared to non-PCOS patients.

Another meta-analysis by Perez-Lopez et al. [38] published in 2021 confirmed the observation that patients with PCOS are characterized by higher serum kisspeptin levels. It has since become increasingly common to look on Kisspeptin as diagnostic marker of PCOS [39].

Esparza et al. [40] studied the neuroendocrine basis of PCOS using mouse models. They observed that LH pulsatility was increased in PCOS; however, the main conclusion from their study was that increases in kisspeptin and neurokinin B expression at the level of arcuate nucleus was likely the key contributor to this change in pulsatility.

Katulski et al. [41] studied kisspeptin and gonadotropins (FSH, LH) pulsatility in patients with PCOS. They found that spontaneous episodic kisspeptin secretion was coupled with LH pulses, but only in eumenorrheic patients. Oligomenorrheic PCOS patients where not found to have coupling of kisspeptin pulses with LH pulsatility. Additionally, PCOS patients with oligomenorrhea were found to have an increased kisspeptin pulse frequency. This study confirmed the hypothesis that disturbances in neuroendocrine function (as seen in PCOS) influence the coupling of kisspeptin with LH pulses. This is a fundamental step in understanding the evolution of a complex syndrome.

The role of kisspeptin as it relates to the metabolic changes observed in PCOS cannot be understated. Obesity, insulin resistance, and hyperlipidemia, are all commonly observed in this syndrome. In a study by Wang et al. [42], kisspeptin levels were found to correlate negatively with glucose area under the curve (AUC), insulin AUC, and triglyceride levels. Similar findings were published by Rashad et al. [43] where serum kisspeptin levels were found to be higher in PCOS patients. Kisspeptin levels were found to decrease with increasing BMI and were negatively correlated to glycemic and lipid profile.

It is presumed that elevated serum kisspeptin levels in PCOS cause hypothalamic–pituitary–gonadal axis overactivity. In turn, this causes menstrual disorders, hyperandrogenemia, and hyperandrogenism. Future investigations are required to understand the precise mechanisms and role of kisspeptin in the pathophysiology of PCOS.

## 6. Neurokinin B and PCOS

Despite PCOS being the most common endocrinopathology in women of reproductive age, its pathophysiology is still not fully understood [44,45]. Many studies focused on neuroendocrine components of the syndrome which are known to play a major role in PCOS etiology. Recent investigations have reported on the abnormal activity of hypothalamic GnRH activity, which is believed to be involved in this process, with a specific focus on the pulse frequency and amplitude of GnRH secretion [45]. GnRH neurons, which are located in the hypothalamus, play a vital role in the onset of puberty and ovarian function [46].

GnRH secretion is regulated by a number of neural and endocrine factors. NKB is one of the key contributors [47]. It is a member of the tachykinin family of peptides, which share a common C-terminal amino acid motif (Phe-X-Gly-Leu-Met-NH2). In humans, NKB is encoded by the *TAC3* gene and binds preferentially to the neurokinin 3 receptor (NK3R; encoded by the *TAC3R* gene). NKB and NK3R are present throughout the neural system in human [48]. The first insight into the role of NKB as a modulator of reproductive function was reported in a study on premenopausal and postmenopausal women in 1991 [49]. NKB is considered a major regulator of GnRH secretion. Clinically, the action of NKB manifests as an increase in thecal androgen secretion and decrease in ovulation ratio, a presentation that is typical for polycystic patients [50]. NKB regulates gonadotropin secretion, follicular development, and the timing of ovulation in healthy women [51].

Unfortunately, little is known about the dysregulation of the KISS1 and NKB systems as it relates to the ovary of PCOS patients [48]. The pathomechanism underlying POCS is very complex and includes developmental factors, metabolic factors (e.g., hyperinsulinemia), and genetic factors.

NKB and kisspeptin signaling is involved in estrogen negative feedback regulation, which itself has been shown to be altered in PCOS. Furthermore, PCOS is associated with an increase in LH pulse amplitude and pulse frequency, which is likely caused by an increased pulsatile secretion of GnRH [52].

Increased pituitary secretion of LH results in failure of ovulation and an increase in ovarian testosterone production. Recent studies suggest that the NKB-kisspeptin-GnRH pathway is crucial in regulating LH secretion. It was found that patients with genetically impaired NKB signaling have lower LH secretion and a decreased LH pulse frequency. Therefore, it was concluded that pharmacological NKB blockade may be a useful approach in targeting the central pathophysiology of LH hypersecretion and hyperandrogenism seen in PCOS.

While the potential contribution of kisspeptin and NKB in PCOS is assumed to be mainly central acting, recent studies show that locally deregulated expression of KISS1 and NKB at the level of granulosa cells may also contribute to the disruption in gonadal to central feedback of the hypothalamic-pituitary-ovarian axis that is characteristic in PCOS [53].

In a study conducted by Blasco V. et al., it was found that levels of NKB and NK3R mRNA were decreased in mural granulosa cells (MGCs) and cumulus oophorus cells (CC) in PCOS women. Moreover, it was found that NKB positively correlated with KISS1 in MGCs of healthy women, whereas no such association was observed in women with PCOS. In healthy women, the transition between MGC and CC is accompanied by upregulation of the TAC3/TACR3 system, a transition which does not occur in PCOS [53]. These findings clearly suggest that that interruption in NKB secretion can contribute to the development of PCOS. Nevertheless, future studies are needed to determine the precise mechanism of the NKB pathway and its contribution to PCOS.

## 7. Kisspeptin and NKB Analogs in PCOS

Kisspeptins are potent and effective stimulators of the release of endogenous GnRH; they can, therefore, provide a more physiological gonadotrophin stimulus to the ovary. As such, kisspeptin-based protocols have been proposed as a safer option for promoting oocyte maturation and ovulation.

A therapeutic receptor agonist specific to the kisspeptin receptor (KISS1R) could significantly expand the potential clinical utility of the kisspeptin pathway [54].

It has been shown that a single injection of kisspeptin-54 (KP-54) is effective in stimulating oocyte maturation in women undergoing in vitro fertilization therapy [55].

Women with PCOS are particularly vulnerable to ovarian hyperstimulation syndrome (OHSS) when undergoing fertility therapy. A recent study by Abbara, A. et al. demonstrated a substantial decrease in associated risk of OHSS in high-risk women with the use of kisspeptin-54.

In the study, 71 women were administered kisspeptin-54. The criteria of inclusion to the study were: age 18–34 years; both ovaries intact; serum AMH at least 40 pmol/L (5.6 ng/mL) or total antral follicle count (AFC) greater than 23, body mass index (BMI) 18–29 kg/m2, and early follicular phase serum FSH less than or equal to 12 mIU/mL. In total, 95% of subjects presented with oocyte maturation. The highest oocyte yield (121%) was observed following administration of 12.8 nmol/kg kisspeptin-54, which was +69% (confidence interval, −16–153%) greater than the next highest dose of 3.2 nmol/kg. At all administered doses of kisspeptin-54, biochemical pregnancy, clinical pregnancy, and live birth rates per transfer (*n* = 51) were 63%, 53%, and 45%, respectively. No woman developed moderate, severe, or critical OHSS. Thus, kisspeptin-54 demonstrated promise as a novel approach to triggering effective and safe oocyte maturation in women undergoing in vitro fertilization (IVF) treatment and who are at high risk of developing OHSS [56,57].

Recent studies have further explored the possibility of using KP-54 for the stimulation of ovulation in patients with PCOS. Romero-Ruiz A. et al. conducted a study to assess the efficacy of KP-54 in stimulating ovulation. Hormonal and ovulatory responses to repeated KP-54 administration, at doses of 6.4–12.8 nmol/kg, subcutaneous injections twice a day (s.c. bid) for 21 days, were evaluated in a pilot cohort of anovulatory women (*n* = 12) who were diagnosed with PCOS as per Rotterdam criteria. Administration of kisspeptin-54 resulted in a significant rise in LH (before kisspeptin-54 administration 10.8 ± 1.8 and after kisspeptin-54 administration 13.4 ± 1.6 IU/L, *p* = 0.038), with corresponding increase in serum estradiol (before kisppeptin-54 administration 113 ± 9.7 and after kisspeptin-54 administration 148.6 ± 11.9 pmol/L, *p* = 0.029). Two women presented with dominant follicle growth with a subsequent ovulation, one woman showed follicle growth without rupture of follicle and ovulation. Despite its low sample size the pilot study succeeded in its objective as a proof-of-principle model and signals promising results for PCOS patients. This first-in-human study demonstrated that KP-54 administration in anovulatory women with PCOS can stimulate reproductive hormone secretion and ovulation, albeit with incomplete efficacy. [57].

A similarly diverse set of outcomes was observed in our first-in-woman, pilot study (*n* = 7). Repeated administration of KP-54 in a group of anovulatory women with PCOS demonstrated a modest, albeit significant, integral LH and estradiol responses in a majority of individuals, with successful induction of ovulation in two out of seven patients. While the limited number of women included in this study limits extrapolation to more general conclusions, the parallels with our preclinical data reinforce the potential value of kisspeptin stimulation as a viable management option for the treatment of anovulatory PCOS.

Increasing the frequency of GnRH pulsation (and, therefore, LH pulsatile secretion), while avoiding impacting FSH secretion is central to the pathophysiology of PCOS. Suppression of the stimulatory role of kisspeptin by specific receptor antagonists and decreasing GnRH secretion may be a therapeutic target in such patients. It is proven that GnRH pulse frequency stimulates LH secretion in a much more profound manner when compared to FSH; a decrease in GnRH pulse frequency has the potential for normalization of LH hypersecretion, often seen in PCOS. Normalization of LH secretion and subsequent normalization of androgen concentration in PCOS may promote folliculogenesis and ovulation [58]. So far, kisspeptin antagonists are not used in this indication, but NKB antagonists are widely used in this situation in clinical research.

In a study conducted by George, J. et al., pharmacological intervention with AZD4901 (a specific NK3 receptor antagonist) was initiated in patients with PCOS for a duration of 28 days. On day 7, patients receiving the neurokinin B antagonist at a dosage of 80 mg/day were found to have a reduction in LH concentration of 52.0%, a reduction in total testosterone concentration of 28.7%, and a reduction in LH pulses to 3.55 per 8 h. The NK3 receptor antagonist specifically reduced LH pulse frequency and subsequently serum LH and T concentrations; a promising potential approach to treating the central neuroendocrine pathophysiology of PCOS [52]. Skorupskaite K et al. demonstrated that administration of a neurokinin 3 receptor antagonist reduced LH secretion (4.0 ± 0.4 vs. 6.5 ± 0.8 IU/L, *p* < 0.05); this study also observed a reduction in FSH secretion (2.0 ± 0.3 vs. 2.5 ± 0.4 IU/L, *p* < 0.05) [59].

## 8. Other Neurohormones and Adipokines in PCOS (Phoenixin-14, Galanin, GLP-1)

There is a growing body of evidence that suggests that phoenixin-14, galanin, and GLP-1 have favorable effects in counteracting the progress of PCOS. Phoenixin (PNX) is a newly discovered peptide produced mainly in the hypothalamus by proteolytic cleavage of a small integral membrane protein, and is detected in various tissues including the hypothalamus, pituitary, heart, gastrointestinal lining, pancreatic islets, and adipose tissue [60]. In vitro studies of anterior pituitary cells found that phoenixin may up-regulate secretion of pituitary gonadotropins, including FSH and LH, by modulating the expression of the gonadotropin-releasing hormone receptor and further by potentiation and up-regulation of GnRH receptors [61]. Moreover, PNX is shown to stimulate insulin secretion, indicating that it may play a role in controlling glycaemia by interacting with pancreatic beta cells [62]. A recent study into the effects of PNX on reproductive function found it to be a compelling intraovarian factor. By accelerating proliferation of human granulosa cells and inducing estradiol secretion, it was shown to stimulate follicular cell development [63]. In a rodent study conducted on rats with PCOS, increased levels of serum PNX was observed, confirming previous observations in the study of patients with PCOS. The concentration of serum PNX-14 was significantly higher in subjects with PCOS than in control groups. This increase correlated positively with testosterone and LH levels and negatively with estradiol (E2). It is thought that increased phoenixin-14 expression in PCOS patients is associated with up-regulation of LH and androgen production [64].

On the other hand, galanin is a neuropeptide widely distributed throughout the central and peripheral nervous system as well as the reproductive tract [65]. It is produced in the hypothalamus and anterior pituitary where its synthesis is highly stimulated by estrogens. Galanin controls preovulatory surges of LH and prolactin and regulates steroidogenesis in ovarian tissue as an intraovarian regulatory peptide. It can be co-secreted with gonadotropin-releasing hormone (GnRH) and can modulate its secretion [66]. Galanin also has a modulating effect on steroid hormones: it can significantly reduce the levels of LH, insulin, glucose, insulin resistance (IR), and testosterone, and stimulate secretion of FSH. It may have a regulatory role in metabolic, inflammatory, and hormonal disorders, as well as gene expression in PCOS. This may imply that galanin holds a potential target pathway which can be used for treatment of PCOS in the future.

Glucagon-like peptide-1 receptor agonists (GLP-1 RA) are a family of glucose-lowering medication having incretin mimetic action. They have been licensed for the treatment of type 2 diabetes mellitus and, more recently, in the management of conditions such as PCOS. Incretins, however, are gut hormones released by enteroendocrine cells that stimulate pancreatic insulin secretion. GLP-1 RA treatment increases accessible plasma GLP-1 to supraphysiologic levels, increasing glucose-dependent insulin secretion, decreasing glucagon production, slowing gastric emptying, and increasing satiety [67].

The few trials that have been conducted on GLP-1 RA in PCOS have all been brief in length and have all demonstrated improvement in glucose and metabolic parameters with varying findings in regard to fertility and hyperandrogenism [68].

Recent studies in induced rat models have shown that the expression of GLP receptors in the hypothalamus, pituitary, and ovary change during the ovulatory cycle and treatment with natural GLP-1 quadrupled the amplitude of the LH surge and resulted in higher progesterone in the luteal phase [69].

## 9. Neurotransmitters in PCOS

In the case of PCOS etiology, it is important to pay attention to the structure and functionality of the underlying neurotransmitter profile. As mentioned previously, the impaired pulsatile secretion of the gonadotropin-releasing hormone (GnRH), and dysregulated hypothalamic-pituitary-ovarian axis are commonly considered the key features underlying the pathophysiology in this syndrome, an implication which suggests strong associated neurotransmitter activity. Neurotransmitters such as GABA, glutamate, serotonin, dopamine, and acetylcholine, as well as the opioid system, may interfere with secretion of GnRH.

Gamma-aminobutyric acid (GABA) is the main inhibitory neurotransmitter in the central nervous system (CNS). The influence of GABA in the pathogenesis of PCOS is widely accepted as it exhibits an excitatory effect on GnRH secretion. Silva et al. proved that GABA neurons derived from the arcuate nucleus stimulate the secretion of GnRH and lead to a robust response of luteinizing hormone (LH) secretion [70]. In similar works, it was found that women diagnosed with PCOS had elevated levels of GABA in cerebrospinal fluid compared to eumenorrheic, ovulatory women [71]. Moreover, GABAergic innervation to GnRH neurons is considered to be responsible for the observed reduced sensitivity to progesterone negative feedback of GnRH and LH pulsatile secretion [72].

As the main excitatory neurotransmitter in the CNS, the role of Glutamate, as it relates to the pathogenesis of PCOS, is still unclear. Kawwass JF. et al. investigated 27 women diagnosed with PCOS and found similar levels of glutamate in cerebrospinal fluid when compared to a control group [71]. Animal model studies, however, have shown elevated glutamate levels and high N-methyl-D-aspartate (NDMA) receptor expression in PCOS—induced rats [73].

The opioid system consists of β-endorphin, enkephalins, and dynorphins-3 families of peptides, whose operation can be divided between central and peripheral systems. Endorphins are responsible for analgesia, stress response, and emotional processes, as well as reproductive neuroendocrine function. Enkephalins play a role in stimulating insulin release, carbohydrate metabolism, modulate the pathogenesis of obesity, and follicular maturation in the reproductive cycle. Dynorphins are potent peptide hormones which act on a variety of opioid receptors, both centrally and peripherally, and contribute to a wide variety of downstream effects.

Beta-Endorphins, peptide hormones which act as opioid receptor agonists, are found widespread in the CNS, but also in pancreatic islets where they have a stimulatory effect on insulin and glucagon release and lead to elevation of glucose levels in the serum [74]. Kiałka M. et al., in their study, observed increased levels of β-endorphins in serum in patients with PCOS compared to the control subjects, these findings also correlated with increased insulin levels and decreased SHBG. As obesity alone has a known impact on β-endorphin levels, the study included only lean, non-obese women [75]. Studies conducted on the use of opioid antagonists, naltrexone and naloxone for the treatment of PCOS, found that this kind of therapy significantly reduced the insulin response to oral glucose tolerance test (OGTT) in a group of hyperinsulinemic patients with PCOS [76]. Naltrexone was found to both ameliorate clinical features such as acne, hirsutism, and amenorrhea, but also to decrease serum androgen and insulin levels while restoring sensitivity to clomiphene citrate in resistant patients [77].

Acetylcholine (ACh) is the main transmitter of the parasympathetic nervous system and should not be overlooked when considering the development of PCOS. Parasympathetic nerve signaling to the ovaries is supplied mostly by the vagus nerve. A study by Linares, R. et al. performed on rats with estradiol valerate injection-induced PCOS, that underwent unilateral or bilateral vagus nerve section, revealed that this procedure can restore spontaneous ovulation in both ovaries in 75% of subjects [78]. In a similar study by the same authors, and again using PCOS-induced rats, 700 mg of atropine was administered as a competitive antagonist of the muscarinic acetylcholine receptor. As in their previous study, spontaneous ovulation was observed in over 70% of subjects [79].

The role of monoamine neurotransmitters, such as serotonin, norepinephrine, and dopamine, in the development of PCOS is poorly studied. In the aforementioned rodent study by Linares, R. et al. a significant reduction was observed of all monoamine neurotransmitters in analyzed tissues [73].

Dysregulations in neurotransmitter physiology may also have a strong psychological impact, a common feature in women with PCOS. Some studies have shown that the prevalence of depression in young women affected with PCOS may be as high as 40%, compared with 10% in control group. Affected women are also shown to be at high risk of developing eating disorders (14%) and anxiety (15%) [79].

## 10. Conclusions

Dysregulation of KNDy neurons activity, as well as abnormal secretion of other neurohormones and neurotransmitters, may contribute to dysfunction of GnRH secretion, thus increasing FSH and LH secretion and development of PCOS. Profound knowledge on this process may contribute to development of new approaches to the treatment of PCOS patients. In the future identification, other specific neurons and signaling factors altered in PCOS may be used to develop potential therapeutics. Moreover, studying the role of metabolic sensitive neurons (for instance AgRP/NPY and POMC) may have potential in explaining neuroendocrine determination of PCOS syndrome.

## Data Availability

Study did not report any data.
